# Gender integration of agricultural innovation: implications for the genetically modified crop product development pipeline

**DOI:** 10.1080/21645698.2024.2431203

**Published:** 2024-12-07

**Authors:** Elizabeth Katz

**Affiliations:** a Department of Economics, University of San Francisco, San Francisco, CA, USA; b Global Center for Gender Equality, Washington, DC, USA

**Keywords:** Crop innovation, crop trait preferences, gender analysis, gender integration, genetically modified crops

## Abstract

We provide guidance on how to incorporate best practices around gender integration in the development of genetically improved crops by adapting a gender integration framework for conventional crop breeding to the GM product development pipeline, which places greater emphasis on the discovery and launch phases because the technical nature of the development process means fewer opportunities for farmer engagement or pivoting possibilities between these two ends of the product development spectrum. For crop innovation to be relevant to both women and men producers, during the discovery phase, developers can conduct baseline gender analysis consisting of gender-disaggregated value chain analysis, systematic learning about gender-specific crop trait preferences, and identification of varietal preferences by women and men along the value chain. The latter opportunity in the GM product development pathway for intentional gender integration is deployment, including pre-launch activities such as field demonstrations and consumer testing. We also describe ex ante and ex post gender impact assessment methods. We conclude with a number of gender integration recommendations for GM product developers: improving gender data collection and analysis to inform crop innovation efforts, investing in staffing and training of scientific teams to enhance gender expertise, and increasing accountability of product development teams with gender-intentional monitoring and evaluation systems.

## Introduction

A new generation of crops being developed for use in low- and middle-income countries uses genetic modification (GM) to enhance productivity and nutritional value, improve pest resistance, and facilitate climate change adaptation. The development of these improved crops shares some characteristics with traditional crop breeding, but also has some unique features that may be important for optimizing their adoption by and generating benefits for both women and men smallholder farmers. In this paper, we provide guidance about how to incorporate best practices around gender integration in the development of genetically improved crops. We adapt a gender integration framework for conventional crop breeding to the GM product development pipeline, which places greater emphasis on the discovery and launch phases. We conclude with a number of gender integration recommendations for GM product developers.

### Gender in Conventional Crop Breeding: A Brief Background

Public-sector breeding programs that focus on agricultural research for development are changing rapidly. Historically led by breeders, modern approaches are more demand- and market-led, placing producers, processors, and consumers – in other words, clients – at the center of priority-setting and decision-making. It is therefore critical in demand-led breeding to understand client needs, which in turn requires more robust social research methods to realize the market intelligence needed to inform product profiles.^1,2^

For demand-led breeding programs to develop accurate and impactful product profiles, they must use methodologically robust and systematic approaches to capture trait preferences.^3^ Mounting evidence shows that distinct use types and social identities, such as gender, shape trait preferences and varietal adoption.^4^ Trait preferences vary in relation to socio-cultural context and modes of production and processing^5^ and follow gender divisions of labor and market access, with documented differences in preferences between men and women across crops and contexts.^6,7^ For example, desirable cereal crop traits mentioned only by women via various elicitation methods include adaptation to a diversity of growing conditions, tall height for ease of harvest, storage life, ease of dehulling and threshing, quantity of usable flour, cooking time, taste and grain color. Men, meanwhile, uniquely expressed preferences for pest resistance, adaptation to intercropping, and yield per hectare.^7^ Neglecting these trait preference differences risks investing in the development of new varieties that do not appeal to both women and men farmers, thereby contributing to lower adoption of improved varieties among women producers in particular.^8^ The traits a breeder prioritizes in developing a new variety powerfully affect who benefits from the variety and how, yet gendered trait preference analyses are relatively scarce,^9^ leading to a data dearth.^1^

### A Gender and Breeding Framework

Answering the question of *how* to integrate gender into crop-breeding programs and projects has gained momentum over the past 5 years. The most notable effort is the CGIAR Gender and Breeding Initiative (GBI), which emerged after 2016 as a collective of plant breeders, social scientists, and gender specialists to develop approaches and strategies for gender integration. The GBI identified key questions to guide segmentation and targeting, understanding trait preferences, priority-setting, and testing and selection.^10^ Polar et al.^9^ expanded this approach and mapped it onto a generalized breeding cycle to demonstrate critical decision points, such as the development of the customer and product profiles, genotype selection, delivery strategy, and monitoring and evaluation. This latter framework, which includes an impact pathway from breeding for development to gender equality as well as gender-related interventions and outputs along the breeding cycle, is the one we adapt for use by GM crop innovation.

### Gender Integration in the GM Product Development Pipeline

These frameworks can be adapted to the GM product development pipeline, which consists of six phases: (0) Discovery, (1) Proof of Concept, (2) Elite Event Selection, (3) Regulatory Studies, (4) Pre-Launch, and (5) Launch. Compared to conventional breeding, the emphasis on gender intentional design in GM product development is most critical at the discovery or design stage (Phase 0), and the pre-launch and launch stages (Phases 4 and 5) because the technical nature of the development process means fewer opportunities for farmer engagement or pivoting possibilities between these two ends of the product development spectrum.

#### Phase 0 Discovery: Baseline Gender Analysis

The discovery phase of GM crop development encompasses two key decisions where information about gender differences matter: trait identification and varietal selection (in the case of the latter this is particularly for perennial or vegetatively propagated crops where subsequent trait introgression may be more difficult or impractical). In order to generate meaningful data to inform this phase, a baseline gender analysis should be conducted, consisting of three components: (1) gender-disaggregated value chain analysis, (2) systematic learning about gender-specific crop trait preferences, and (3) identification of varietal preferences by women and men along the value chain. We describe each of these in turn.

#### (1) Gender-Disaggregated Value Chain Analysis

The first step to learning about women’s and men’s roles in the production and distribution of a crop under consideration for genetic modification is to generate a value chain map that shows the movement and relative volume of the target crop in a specific country, layered with a gender analysis of how women are positioned in that product value chain, the result of which would be a gendered value chain map.^2^^11–13^ This may require both accessing secondary sources and conducting primary fieldwork, and it necessitates collaboration with social scientists, including gender experts.^3^ During field research to construct the gendered value chain map, qualitative interviews with respondents from across the value chain could be added for a deeper dive on potential gender-based constraints and opportunities for women and men in a new GM product introduction into the existing value chains and markets.^4^^,^^5^ This value chain analysis provides an initial evidentiary basis for the gender division of labor in the target crop, which in turn informs the potential trait preferences of women and men, depending on their roles in production, processing, marketing, and consumption.

#### (2) Systematic Learning About Gender-Specific Crop Trait Preferences

Building on the gendered value chain analysis, GM product development teams can dedicate resources to systematically eliciting and evaluating end-user trait preferences along the value chain, by gender. While genetic modification can broaden the scope of trait selection, as it is not restricted to crosses between sexually compatible species as with conventional breeding, GM product development tends to focus on one or potentially a stack of two to three complementary traits for transformation (although this is changing in light of technological advancements within genome editing). A large body of literature documents men’s and women’s different, and overlapping, trait preferences across multiple crops.^6^ It is therefore essential to conduct a trait preference study at an early stage in the GM product development pipeline that includes gender analysis to understand where, how, and why trait preferences may differ between men and women.^7^^,^^8^

To assess whether the GM product to be released will have a positive benefit for women and men, the trait preference study should verify that the agronomic production or quality trait (e.g., weed management, pest management, cooking time) is salient for farmers of both genders. Relatedly, if baseline research identifies any potential negative impact from the choice of traits – for example, displacing women from crop value chains through commercialization of the released GM product – then this should be a pause point before proceeding to Phase 1.

#### (3) Identify Varietal Preferences by Women and Men Along the Value Chain

A third entry point for gender integration during the design phase is in the choice of germplasm for transformation. The choice of varietal background for the transformation event(s) is typically based on a combination of varietal preference in a target country or region and its amenability to genetic transformation. A significant body of evidence shows that women and men may make different choices in adopting conventional crop varieties, and for different reasons.^14,^^15–17^ For example, in Uganda, women plot managers are significantly less likely to adopt both drought-tolerant and non-drought-tolerant maize varieties if they frequently experienced climate shocks and dry spells during the growing season, providing additional support for the hypothesis that women farmers are more risk-averse on average and therefore less inclined to adopt new technologies when they experience shocks.^18^

In defining market relevance, programs should base these decisions on robust varietal preference studies that consider social heterogeneity in sampling, and present results to show differential varietal preference by sex, age, and other relevant variables. After this gender analysis of varietal preferences, the program can make decisions on which varieties to consider, positively selecting for those that both women and men prefer in order to increase the potential rate of adoption of the resulting product among producers of both genders. Depending on how readily trait(s) can be introgressed into other plant varieties, programs should proactively consider those varieties that should be used for trait introgression after the lead event is selected for advancement, as there is emerging literature on the importance of varietal choice for women farmers as a path to empowerment.^9^

Phase 0 gender analyses are even more critical for GM crops than conventional breeding, as there is limited opportunity to course-correct or build in inclusive and participatory selection steps during Phases 2–3 because of the highly structured and costly approach required for GM product development. Keeping in mind that there may be limitations on how readily trait(s) can be introgressed into other plant varieties, choosing the wrong germplasm for transformation or selecting a trait that is not valued by both women and men is potentially more consequential with GM than with conventional crops.

### Phases 4 and 5: Pre-Launch and Launch

The second opportunity in the GM product development pathway for intentional gender integration is deployment, including pre-launch activities such as field demonstrations and consumer testing.

Much like the technology development process itself, the scaling of technologies is highly gendered. There is ample documentation that the disparities in access to resources between men and women shape their access to, and benefit from, new technologies. After formal release of a GM product, the seed system into which it is integrated will determine the ability of women and men to access the product.^9^ Seed systems are highly gendered for food security crops, with women often engaging in informal seed systems, while men are more able to benefit from formal seed systems, necessitating synergies between the two to improve availability and accessibility of quality seeds for both women and men.^19^ Balancing the interventions to engage informal seed systems would increase the chances of women benefiting from these interventions (including productivity and time use gains) and have a multiplier effect on outcomes such as enhancing smallholder household food security and nutrition from GM product development.

Much like the baseline gender analysis described above for Phase 0, it is critical during the development of launch strategies (developed before Phases 4 & 5) to identify gender-based constraints and gaps in access to seed. These strategies should be reevaluated so that constraints that will affect deployment and scaling can proactively be addressed. Complementary interventions that enhance the inclusiveness of seed dissemination include vouchers, bundled services, and a focus on how extension information is delivered, given receptiveness to new information,^20^ all of which should be considered in the dissemination strategy.

All demonstration, marketing, dissemination, and training activities should be gender responsive and consider the ways with which women and men differentially engage with knowledge, resources, and training. Training programs for farmers and seed entrepreneurs should ensure the training takes place at the right time and location to ensure that both women and men can participate, and planned trainings should be completed with consultation from a gender specialist engaged in the programs. The dissemination stage of the Pod Bore Resistant (Bt) cowpea development program in Nigeria, for example, made a concerted effort to reach women farmers, processers, and consumers, by setting a goal of 30% participation in the national performance trials (NPTs), and actively recruiting women into the consumer testing, wherein popular cowpea dishes were cooked using the PBR cowpea for people to taste and demonstrate that it tasted similar to conventional cowpea.^21^

Box 1 summarizes the key gender integration priorities for GM product development.

Box 1. Key gender integration actions for GM product development

- Identify and select a market segment where the GM product will be released; ensure that it has a high proportion of women and men producers, processors, and consumers who would benefit
- Prioritize germplasm for transformation based on varieties that are highly valued and preferred by women and men producers
- Carefully validate that the trait(s) chosen for transformation do not unintentionally do harm
- Ensure that baseline gender analysis that precedes product development is budgeted for, and supported by, in-country partners
- Conduct gender analysis of seed systems for target countries that precedes launch of the GM product
- Design dissemination strategy that builds on the gender analysis of seed systems in each target country and includes both formal and informal seed system networks
- Ensure that all technology, policy, marketing, and training activities carefully consider gender
- Engage well-qualified and experienced gender expertise throughout the GM product development process
- Include robust sex-disaggregated data collection in the program monitoring, evaluation, and learning agenda, aiming to include clearly articulated gender- and social-inclusion-specific indicators and metrics, and aiming to evaluate the GM product’s impact toward gender equality and social-inclusion goals

### Gender Impact Assessment Methods

Diverse breeding efforts call for research prioritization and robust gender-impact studies.^22^ This is particularly true in the case of GM crops, where the financial resources and time spent in product development justify attention to *ex-ante* and *ex-post* evaluations of the new technology.^23^ *Ex-ante* and *ex-post* impact assessments are important informational tools in the development of new GM products, but gender dynamics are frequently overlooked in these exercises.^10^ In this section, we provide several high-level methodological considerations for how gender integration should be included in *ex-ante* and *ex-post* GM crop evaluations.

#### Phase 0: Ex-Ante Evaluations

Evaluating ex-ante impacts of GM crops serves two purposes: for plant breeders to meet users’ needs, it helps uncover the priorities that women and men assign to crop traits^24^; and for breeding programs to be impactful, it supports the elaboration of rankings which reflect the heterogeneity of priorities across different disciplines and experts, such as plant breeders, gender specialists, rural development experts, agricultural economists, and value chain stakeholders. The emerging literature on ex-ante frameworks for crop priority-setting and impact assessments can be informative for GM crop impact studies.^25–28^

To effectively integrate gender into ex-ante evaluations, the focus on monetized costs and benefits needs to be broadened to encompass externalities, distributional effects, and longer-term impacts^29,30^. Unlike yield, crop characteristics that are disproportionately valued by women, such as ease of peeling, higher nutrient content, culinary attributes, richness in vitamins, and

postharvest qualities, are less easily assigned monetary values. Alternative methods, such as weighted criteria approaches and scoring models, in conjunction with techniques for data integration and data gamification, may help to incorporate gender analysis. These methods also contribute to a priority-setting framework that facilitates the elicitation of stakeholders’ preferences, while accounting for the multidimensionality of the prioritization. This requires accounting for (i) emergence of significant trade-offs, so priority-setting requires parametrizing weights to each research priority; and (ii) necessity to aggregate different objectives to obtain a final score for each alternative.

#### Post Phase 5: Ex-Post Evaluations

Gender -informed ex-ante evaluations should be complemented with robust ex-post evaluations. Compiling ex-ante data and estimated parameters on relevant indicators (across the economic, breeding, environmental, gender, and climate domain) is a relatively costly, but extremely useful exercise for GM breeding teams. It creates a robust set of baseline data to which endline estimations can be easily compared. Whenever ex-post evaluations occur detached from ex-ante destinations, researchers working with GM crops should pay attention to two elements: first, the presence of varietal fluctuations in adoption studies and second, the design of ex-post evaluation methods (keeping the randomized control trial method as the gold standard) which account for gendered impact pathways. In this section, we briefly describe both points.

Heterogeneity in varietal preferences of adopted crops is usually explored with a cross-sectional lens (e.g.^31^ Varietal preferences are generally not monitored over time, and it is very rare that the same respondents are asked longitudinally whether their trait preferences have evolved. Overlooking varietal fluctuation in adoption studies – especially GM adoption studies – might lead to very low levels of adoption. Ignoring what is frequently defined as a varietal age might lead to a partial picture of the array of reasons why a GM crop is adopted and persists to be adopted over time. Even when a new variety looks highly promising for many traits, a single negative trait can lead to non-adoption).^32^ Removing that single negative trait is possible only through further lengthy breeding.

There are several examples of sex-disaggregated ex-post impact evaluation of GM crops in low and middle-income contexts, many of which focus on changes in labor allocation.^33^ Gouse et al.^34^ examine the gender-specific adoption and performance effects of insect resistant (Bt) and herbicide-tolerant (HT) maize produced by smallholder farmers in the Kwa Zulu Natal province in South Africa and find that women farmers value the labor-saving benefit of HT maize, while yields are the main reason behind male adoption. In contrast, in India, it was found that female laborers benefited from the increased work hours – and thus increased income – associated with increased yields from *Bt* cotton because women pick the cotton. Conversely, male laborers generally spray chemicals, so they saw a reduction in their labor time.^35^ In Burkina Faso, fewer insecticide applications were needed for *Bt* cotton and that meant women spent less time in fetching water.^36^ In Bolivian households that adopted HR soybean, women had more time to work off the farm.^37^ Female farmers in Colombia who adopted *Bt* cotton preferred IR varieties because they reduced the number of laborers needed, whereas men reported that *Bt* cotton increased yields and overall benefits.^38^ In contrast, HR cotton in Colombia resulted in the hiring of fewer women for weeding, traditionally a female task.^36^ Female maize farmers in the Philippines, whether they grew *Bt* varieties or not, reported that *Bt* saved labor, but men who planted maize did not note a time-saving aspect to either *Bt* or non-*Bt* varieties.^36^

Collecting ex-post longitudinal data on adoption is becoming a necessity for breeding teams. In this context, new decentralized participatory varietal selection methods – such as the triadic comparison of technologies or *tricot*^39^ – can provide a relatively less costly solution to repetitive rounds of adoption surveys. Nonetheless, the tricot method needs to be improved to be gender inclusive, starting from the very first step of the sampling frame. Being a relatively new breeding approach, GM crops are well suited to randomized control trials (RCT), which could incorporate existing quantitative tools, such as the Women’s Empowerment in Agriculture Index, to measure the impact of new crop technologies on women’s and men’s production and income, time allocation, and other direct and indirect outcomes.^11^

### Gender Integration Recommendations for GM Product Developers

Global and national organizations involved in modern crop innovation have the opportunity to advance gender-inclusive approaches, building on, and learning from, the significant progress that has been made in conventional crop breeding in recent years. There are three key areas to focus gender integration efforts: improving data collection and analysis, investing in staffing and training of scientific teams, and increasing the accountability of product development teams with well-designed and managed monitoring and evaluation systems.

#### Improving Gender Data Collection and Analysis to Inform Crop Innovation Efforts

As detailed above, validated methods exist for collecting and analyzing the kinds of data that are required to make evidence-based decisions, particularly during the earliest stages of product development. However, scientific teams may rely on impression and anecdote, as opposed to systematic assessment of the gender division of labor in the crop value chain and gender differences in crop trait preferences. Breeding programs can make use of proven and cutting-edge methodologies for high-quality gender data collection and analysis – including, for example, the G+ tools for gender-responsive breeding of new crop varieties developed by the CGIAR Gender and Breeding Initiative.

#### Investing in Staffing and Training of Scientific Teams to Enhance Gender Expertise

To guide these data collection efforts – and, equally important, to ensure that the findings are incorporated into trait and varietal selection, as well as the dissemination strategy – public breeding programs are going to need significant technical support, and perhaps some degree of culture change. Even where product development teams include social scientists, dedicated budget for at least one gender expert is key to institutionalizing gender analysis as a legitimate component of the crop innovation process. Global funders can provide support for such specialist staffing, as well as offer professional development opportunities to scientific team members to increase their awareness of the relevance of gender analytic methods to successful product development. This can be achieved through various capacity-building activities, including gender training seminars and workshops. Experiential learning is also key: involving scientific teams in the social science research process, where they can observe the robustness of the data collection and analysis, can help build trust in, and appreciation for, the value of the findings and insights that are generated.

#### Increasing Accountability of Product Development Teams with Gender-Intentional Monitoring and Evaluation Systems

Donors have the ability to incentivize their partners to conduct meaningful gender integration work. The principal means by which funders can hold partners accountable for delivering on gender-related objectives is to build specific, achievable, and measurable results frameworks, and then diligently monitor compliance over the life of a product-development pipeline and provide additional technical support as needed. Such gender-intentional M&E frameworks specifically for agricultural research are in their infancy, but one recent example can be found in Mangheni et al.^40^

By learning from the significant advances in gender integration of conventional crop breeding, and adapting best practices to the unique features of GM product development, researchers and developers have the potential to ensure that future generations of agricultural innovations systematically value and contribute to both women’s and men’s key roles in smallholder food systems.

## Conclusions

This paper provides guidance about how to incorporate best practices around gender integration in the development of genetically improved crops. The guidance is critical because both women and men farmers have low adoption rates of improved seed varieties, in part because women in particular are infrequently consulted about their preferences for the traits they value as producers, post-harvest processors, marketers, or consumers. Moreover, public-sector breeding for development distinguishes itself by explicitly focusing on social-inclusion outcomes, such as gender equality, poverty alleviation, and food security as laid out in the Sustainable Development Goals.

In conventional breeding programs, tools have been developed that help us understand why women farmers are slower than men to adopt new seed and how to improve their adoption rates. To adapt those tools for genetically modified (GM) crop breeding, it is important to underscore that GM breeding is unique in that nearly all critical decisions are made in the earliest stages of the crop development pipeline, when the trait(s) and host variety are selected. The GM development pipeline is significantly different from a conventional breeding pipeline and leaves much less room to course-correct by learning and adapting along the way.

This means that, in order to be effective, the focus of gender integration must be conducted in phase zero, the design stage. It is essential to develop products using validated gender analytic tools. Instead of relying on broad generalizations, researchers can learn about the distinct roles of women and men in crop value chains and which traits are most important to them. The traits that a breeder prioritizes in developing a new variety powerfully affects who benefits from the variety and how, yet gendered trait preference analysis is relatively scarce. There are also significant opportunities for gender integration at the pre-launch and launch stages.

In order to develop new GM crop varieties that meet the needs of both women and men, funders and product developers must invest in improving gender-informed data collection and analysis, appropriate staffing and training of scientific teams, and increasing the accountability with well-designed and managed monitoring and evaluation systems.

## Notes

1. Trait preference data are often not collected from women, or not sex-disaggregated in analyses. A recent scoping review^41^ found that in the published trait prioritization literature in the past 50 years, only 26% of papers had any sex-disaggregation in collection and even fewer in analysis. Of those studies that did collect preference data from both women and men, 84% found sex-based trait preference differences.

2. See Teeken et al.^42^ for an example of a gendered product/value chain map for cassava in Nigeria. This analysis identifies the percentage of women and men who participate in the production, processing, and trade of cassava and cassava products such as gari and fufu.

3. One publicly available data collection instrument that can be used for gender analysis of agricultural value chains is the project-level Women’s Empowerment in Agriculture Index for market inclusion (pro-WEAI+MI), which includes the core pro-WEAI module plus new complementary indicators to investigate barriers to market access and inclusion for different value chain actors.^43^

4. For a description of a participatory ex-ante framework for crop improvement, *see* Occelli et al.^41^

5. Malapit et al.^44^ analyze and compare women’s and men’s empowerment along multiple stages of the value chain – production, processing, and trading – in the abaca, coconut, seaweed, and swine value chains in the Philippines.

6. Methodological and analytical methods for assessing gendered trait preferences include: encapsulation (where one set of gendered trait preferences is a subset of trait preferences for the opposite gender); granularity, where either women or men can provide richer, more detailed information about a specific trait; ranking, where either women or men give a higher or lower order of importance or rank to the same trait; and intensity, where either women or men attach greater importance to a specific trait, as measured by a higher score or the frequency with which they mentioned this trait.^45^ These methods have been mostly used in relation to conventional breeding but can be applied to genetically modified crops.

7. Key to these trait preference studies in conventional breeding are that the studies considered are fairly recent (three to five years); the trade-off analysis; and that they include a research design that has addressed sampling for social diversity in the study design, to ensure inclusiveness, while adding gender analysis with emerging tools (such as G+ analysis tools).

8. In the development of podbore-resistant (Bt) cowpea in Nigeria during the early 2000s, where insect resistance was the key trait of focus, it does not appear that resources were invested in learning about other trait preferences that might have differed by gender. This was a missed opportunity because, unlike with common bean (Phaseolus vulgaris), there is little documented research on gender differences in trait or varietal preferences for cowpea.^21^

9. For example, the newer stacked varieties of maize introduced in the Kwa Zulu Natal province in South Africa were preferred by both male and female farmers and seemed to be in high demand by both groups. However, lack of GM seed availability in the region and poor market access were possible limitations to the adoption and spread of the technology.^34^

10. Most studies on GM adoption impacts, like Klümper & Quaim,^23^ Kranthi & Stone,^46^ and Ahmed et al.,^47^ do not sex-disaggregate impacts and leave gender considerations unexplored.

11. Ahmed et al.^47^ quantify the economic and health impacts of Bt eggplant in Bangladesh utilizing an RCT experiment. The results suggest that the new GM crop led adopters to have higher yield, higher income revenues, and reduced health issues connected to a lower use of fertilizers. Gender-related impacts were not explored.
